# Comparison of quadriceps muscle size and quality in adults with cystic fibrosis with different severities of cystic fibrosis transmembrane conductance regulator protein dysfunction

**DOI:** 10.1177/14799731221131330

**Published:** 2022-11-15

**Authors:** Kenneth Wu, Anna Michalski, Jenna Sykes, Jane Batt, Anne L Stephenson, Sunita Mathur

**Affiliations:** 1 Rehabilitation Sciences Institute, Temerty Faculty of Medicine, 12366 University of Toronto, Toronto, ON, Canada; 2 Toronto Adult Cystic Fibrosis Centre, Department of Respirology, 10071 St. Michael’s Hospital, Unity Health Toronto, Toronto, ON, Canada; 3 Department of Physical Therapy, Temerty Faculty of Medicine, 12366 University of Toronto, Toronto, ON, Canada; 4 Department of Respirology, St. Michael’s Hospital, 10071 Unity Health Toronto, Toronto, ON Canada; 5 Institute of Medical Science, Temerty Faculty of Medicine, 10071 University of Toronto, Toronto, ON, Canada; 6 Keenan Research Centre, Li Ka Shing Knowledge Institute, St. Michael’s Hospital, Unity Health Toronto, Toronto, ON Canada; 7 Institute of Health Policy, Management and Evaluation, University of Toronto, Toronto, ON, Canada; 8 School of Rehabilitation Therapy, Queen’s University, Kingston, ON, Canada

**Keywords:** muscular atrophy, mutation, regression analysis, skeletal muscle, ultrasonography, muscle composition

## Abstract

**Background:**

Cystic fibrosis (CF) is characterized by CF transmembrane conductance regulator (CFTR) dysfunction. CFTR protein is expressed in human skeletal muscle; however, its impact on skeletal muscle is unknown. The objectives of this study were to compare quadriceps muscle size and quality between adults with various severities of CFTR protein dysfunction.

**Methods:**

We conducted a prospective, cross-sectional study comparing 34 adults with severe versus 18 with mild CFTR protein dysfunction, recruited from a specialized CF centre. Ultrasound images of rectus femoris cross-sectional area (RF-CSA) and quadriceps layer thickness for muscle size, and rectus femoris echogenicity (RF-ECHO) (muscle quality) were obtained. Multivariable linear regression models were developed using purposeful selection technique.

**Results:**

People with severe CFTR protein dysfunction had larger RF-CSA by 3.22 cm^2^, 95% CI (1.03, 5.41) cm^2^, *p*=.0049], after adjusting for oral corticosteroid use and *Pseudomonas aeruginosa* colonization. However, a sensitivity analysis indicated that the result was influenced by the specific confounders being adjusted for in the model. We did not find any significant differences in quadriceps layer thickness or RF-ECHO between the two groups.

**Conclusion:**

We found no differential impact of the extent of diminished CFTR protein activity on quadriceps muscle size or quality in our study cohort. Based on these findings, CFTR mutation status cannot be used differentiate leg muscle size or quality in people with CF.

## Introduction

Cystic fibrosis (CF) is a genetic disease resulting in dysfunction of CF transmembrane conductance regulator (CFTR) protein. CFTR protein is widely expressed in multiple body organs, including skeletal muscles.^1,2^ In an animal study, CFTR protein dysfunction was shown to be linked to hyperactivation of the nuclear factor kappa-light-chain-enhancer of activated B cells, a central regulator of inflammation, which was demonstrated to be associated with skeletal muscle atrophy.^1^ In our systematic review and meta-analysis of the literature on limb muscle size and contractile function in adults with CF, we found that thigh muscle atrophy is present in the adults with CF.^3^ Individual studies also showed associations between leg muscle size and lung function,^4^ nutritional status,^5^ and physical activity (PA) level.^4,5^

In addition to muscle size, muscle quality is another way to examine limb muscle structure.^6^ Muscle quality reflects the amount of infiltration of non-contractile tissues such as intramuscular adipose or fibrous tissues.^7^ Greater intramuscular fat and/or fibrosis leads to poorer muscle quality and compromises the ability of the muscle to generate force. Studies on the elderly,^8^ chronic obstructive pulmonary disease,^9^ acutely-^10^ or critically-ill^11^ populations have shown that poorer skeletal muscle quality is associated with muscle atrophy. There are no published studies exploring the relationship between the severity of CFTR protein dysfunction and the extent of impairment in skeletal muscle size and quality in adults with CF.

The objectives of this study were to compare (1) quadriceps muscle size and (2) quadriceps muscle quality, in adults with CF with different severities of CFTR protein dysfunction. It was hypothesized that adults with CF with severe CFTR protein dysfunction would have smaller quadriceps muscle size and poorer muscle quality than those with mild CFTR protein dysfunction, after adjusting for confounders.

## Methods

### Study design

This is a prospective, cross-sectional study. This study was approved by the research ethics board at Unity Health Toronto (REB#17–274) and University of Toronto (protocol #00035576). The study is reported based on the STROBE guideline.

### Participants

People with CF attending the outpatient clinic at the Toronto Adult Cystic Fibrosis Centre, St. Michael’s Hospital, Unity Health Toronto were recruited for this study. Individuals were eligible to participate if they were ≥18 years of age, had a documented diagnosis of CF, and a record of CF genetic mutation by genotyping. People were excluded if they had a history of any organ transplant; known diagnosis of cancer, cardiac, neurological, or musculoskeletal diseases; currently pregnant; use of supplemental oxygen with exertion; unintentional weight loss of >10lbs within 1 month prior to the test day; or use of intravenous (IV) or oral corticosteroids within 3 months prior to the test day. Furthermore, people were excluded if they had a pulmonary exacerbation requiring oral or IV antibiotics within 1 month prior to the test day.^12^ People who were on CFTR modulator therapy were also excluded.

People with CF were categorized into two groups based on the extent of the disruption of the normal ion transport functions of CFTR protein. People with only class I-III CFTR mutations are designated as to have *minimal* function CFTR mutations, while those with at least one class IV-VI CFTR mutation are designated having *residual* function CFTR mutations. Minimal function CFTR mutations result in no or minimal CFTR function, whereas residual function CFTR mutations result in partially retained CFTR function.^13^ Hence, people with minimal function CFTR mutations have a greater reduction in CFTR protein functioning than those with residual function CFTR mutations.

### Study protocol

Ultrasound was used to measure muscle size and quality. Participants were tested by one of two testers (KW or AM) in a single test session conducted during their routine outpatient clinic visit. Prior to the study, the two testers were trained by an experienced researcher to perform muscle ultrasound image acquisition. The mean inter-tester difference was rectus femoris (RF) cross-sectional area (RF-CSA): 0.47 cm^2^, the layer thicknesses of RF, vastus intermedius (VI), and vastus lateralis (VL): 0.004–0.05 cm, and RF echogenicity (RF-ECHO): 14 arbitrary units (AU). The mean inter-rater difference was RF-CSA: 0.26 cm^2^, the layer thicknesses of RF, VI, and VL: 0.006–0.02 cm, and RF-ECHO: 1 AU. Both inter-tester and inter-rater differences fell between 95% limits of agreement.

#### Quadriceps muscle size

**Figure 1.**
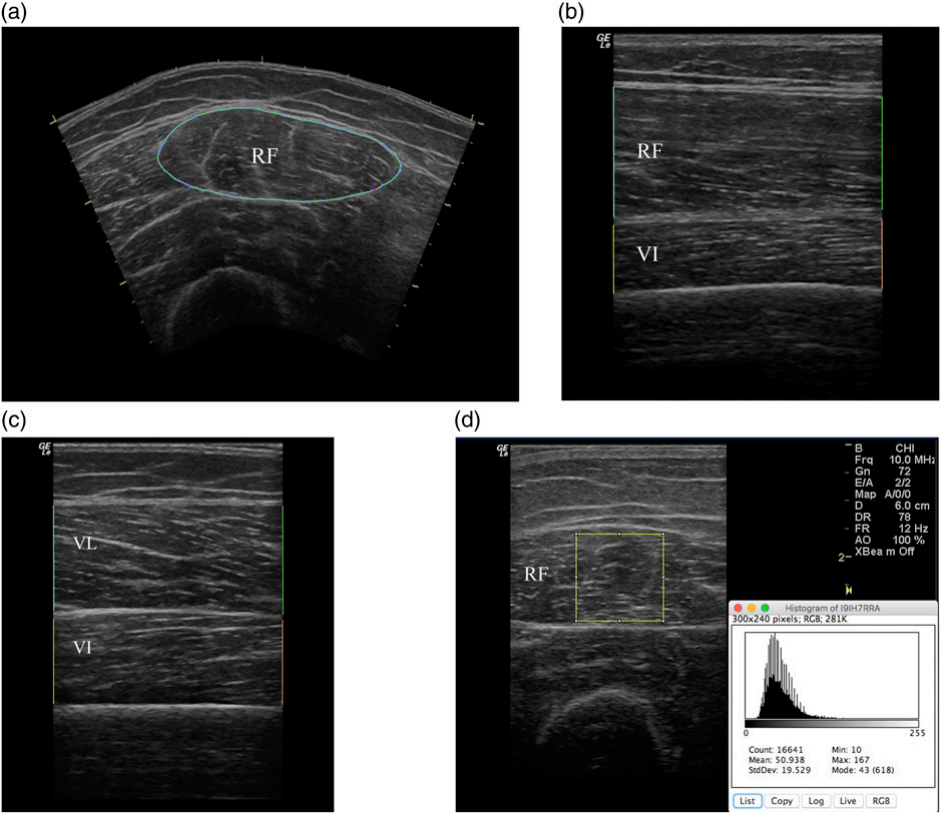
(a) RF-CSA, The RF-CSA was measured by tracing the edge along the inner border of the epimysium (b) Layer thickness of RF and VI anteriorly (c) Layer thickness of VL and VI laterally, and (d) RF-ECHO. A largest possible area within the RF was used to select the region of interest (ROI). Mean greyscale of this ROI was calculated using histogram function. Ultrasound images of the RF-CSA, quadriceps layer thickness, and RF-ECHO Muscle layer thickness was measured at the vertical distance from the superficial to deep aponeuroses. The sum of the thickness of RF, VL and average of VI measured anteriorly and laterally was used as the quadriceps layer thickness. RF Rectus femoris, RF-CSA Rectus femoris cross-sectional area, RF-ECHO Rectus femoris echogenicity, VI vastus intermedius; VL Vastus lateralis.

Quadriceps muscle size (RF-CSA and quadriceps layer thickness) was assessed with B-mode imaging using a GE Logiq E ultrasound system, fitted with an 8–12 MHz linear array probe. All imaging was done on the dominant leg. Participants were positioned in the supine position with knee flexed at approximately 30° resting on a pillow with the hip in neutral rotation. The quadriceps muscle was landmarked at the mid-thigh anteriorly – 50% of the distance between the anterior superior iliac spine and upper superior pole of patella, to obtain images of the layer thickness of RF and anterior aspect of the VI in the sagittal plane. RF-CSA was captured at the same landmark using panoramic imaging mode (extended field of view) in the trans-axial plane. Layer thickness of VL and lateral aspect of the VI was taken at the mid-thigh (50% distance) laterally, at the thickest part of the VL (5.5–10 cm from the midpoint of the thigh). The acquisition parameters were frequency: 8–12 MHz, gain: 56–82 dB, and depth: 4.5–8 cm for RF-CSA; and frequency: 8–13 MHz, gain 56–98 dB, and depth 4.5–9 cm for quadriceps layer thickness. At least three images were captured at each landmark. The images were analyzed using the OsiriX software,^14^ as shown in Figure 1(a) to (c). Quadriceps layer thickness is defined as the sum of the thicknesses of RF, VL, and the average of VI obtained anteriorly and laterally. For each set of measures for quadriceps layer thickness and RF-CSA, the average of three measurements was used for analysis.

#### Quadriceps muscle quality

Muscle quality of the RF was assessed by muscle echogenicity, from the trans-axial images obtained at the anterior mid-thigh (frequency 10 MHz, gain 72 dB, and depth 6 cm). The RF-ECHO was calculated using mean greyscale with the histogram function in National Institutes of Health Image J software.^15^ The region of interest (ROI) was the largest possible square within the anatomic boundaries of the muscle in the centre of the RF (Figure 1(d)).^16^ Mean greyscale of this ROI was expressed as a value between 0 (= black) and 255 (= white), where higher values indicate more fat or fibrous muscle infiltration, i.e. poorer muscle quality.^17^ The average of three measurements of RF-ECHO was used for analysis.

#### Physical activity level

Physical activity level was measured using the 7-day physical activity recall (7D-PAR) questionnaire,^18^ which had been previously validated against tri-axial accelerometery (ActiGraph) in the CF population.^19^ During a semi-structured interview, participants were asked to recall the time they spent on PA with ≥10-min duration at different intensities (moderate, hard, and very hard) and strengthening exercise over 7 days prior to the testing day. Because of the chance of incorrect perceptions of intensity of PA,^19^ the time spent on PA at different intensities was combined for the analysis. As part of the 7D-PAR, participants were also asked about the time spent in exercises that were planned for the purpose of increasing strength.

#### Demographic and clinical variables

A list of potential confounders was developed a priori based on results of our systematic review and meta-analysis,^3^ previous studies in CF, as well as some known associations with skeletal muscles in non-CF studies. They include demographic variables (age^20^ and sex^21^), anthropometric measures (BMI,^5^ body weight,^12,22^ and height^22^), lung function, measured on the testing day [forced expiratory volume in 1 second,^4^ and forced vital capacity (FVC)^4^]; bacterial status (ever colonized with *P. aeruginosa*^23^ and *Burkholderia* species^24^), blood sugar control [diabetes diagnosis^25^ and the most recent hemoglobin A1c (HbA1c) level^25^], vitamin D level,^26^ oral corticosteroid use,^27^ and history of hospitalization (hospitalized in the past 12 months, number of time, and total duration^12^, if applicable), and the time spent on PA or strengthening exercise in the past 7 days.^4,5^ Age of CF diagnosis and pancreatic status were also collected for descriptive purposes. These data were collected from the medical chart, the Toronto CF Database, and the 7D-PAR.

### Statistical analysis

Sample size was calculated using Cohen’s d statistics,^28^ with an anticipated medium effect (*f*^*2*^=0.15, two-sided, type I error = 5%, power = 80%), and assuming six predictors, the estimated sample size was 59 patients, with 60% of the sample from the minimal group.

Descriptive statistics were summarized as median (min-max) for continuous variables; and frequency (proportion) for categorical variables. Demographic and clinical variables were compared between the two CFTR mutation groups with independent *t*-test for normally distributed continuous variables, Mann-Whitney-Wilcoxon test for non-normally distributed continuous variables, and chi-square test for categorical variables.

Multivariable linear regression models were developed for (1) RF-CSA, (2) quadriceps layer thickness, and (3) RF-ECHO. Purposeful selection technique^29^ was used to fit these models. Because BMI and body weight are covariates, two multivariable regression models with either of these two variables in each model were developed. Bayesian information criterion (BIC) were then used to select the final model (the one with the lower BIC score). Age was different between the two CFTR mutation groups (*p*<.25) in the first step of the purposeful selection technique, but only affect the coefficient of CFTR mutation group by >20% in the second step of the purposeful selection technique for the multivariable regression model of RF-ECHO, hence age was adjusted only in the RF-ECHO model. There was no significant difference in sex between the two CFTR mutation groups in the first step of the purposeful selection technique in all regression models. Moreover, interaction of Vitamin D and CFTR mutation group was identified when the relationship between the quadriceps layer thickness and CFTR mutation groups differed above and below the median Vitamin D level of all participants (i.e. the cut-off point).

*Post hoc* analysis was performed to examine the RF-CSA for influential cases with Cook’s distance plot. Influential cases were defined as data that are at or above the cut-off point, which was set at 4/the number of observations.^30^ Sensitivity analyses were performed for RF-CSA using the same confounders in the original model without the influential cases; and RF-CSA of whole dataset using the confounders from the quadriceps layer thickness (another measure of quadriceps muscle size). All statistics were analysed using the open-source software R, version 4.1.0.^31^ All *p*-values are two-sided and assessed at *p* <.05 unless otherwise stated.

## Results

Between April 2018 and mid-March 2020, 450 patients were screened, 171 individuals were eligible and contacted for the study. A total of 52 adults with CF participated in the study (34 in the minimal group and 18 in the residual group). Recruitment and testing for the study were halted in mid-March 2020 because of the COVID-19 lockdown.

The characteristics of participants in the two CFTR mutation groups are summarized in Table 1. People in the minimal group were lower in age (*p* = .0029), more frequently colonized with *P. aeruginosa* (*p* = .033), and had higher HbA1c (*p* = .016), compared to people in the residual group. Only one participant was on IV corticosteroid in the past 12 months from the testing day (not shown in Table 1).

**Table 1.** Characteristics of participants in the two CFTR mutation groups.

	Minimal (*n* = 34)	Residual (*n* = 18)	*p*-value
Age (year)	29 (19–50)	42 (22–66)	**.0029**^c^
Sex (male)^e^	21 (62%)	8 (44%)	.37^a^
BMI (kg/m^2^)	22.7 (17.8–29.4)	24.4 (19.4–32.4)	.27^c^
Body weight (kg)	63.8 (42.1–100.1)	69.2 (48.2–106.0)	.35^c^
Height (m)	1.69 (1.53–1.85)	1.69 (1.53–1.84)	.79^d^
Genetic mutation^e^			
Homozygous F508del	22 (65%)	0 (0%)	
Heterozygous F508del	12 (35%)	12 (67%)	
No F508del	0 (0%)	6 (33%)	
Age of CF diagnosis (year)	0.5 (0–10.1)	23.5 (0.2–52)	
Pancreatic insufficiency^e^	34 (100%)	5 (28%)	
FEV_1_ % predicted^f^	70 (26–104)	74 (29–107)	.34^c^
FVC % predicted^f^	86 (50–117)	96 (42–118)	.19^c^
Bacterial status^e^			
*P. aeruginosa*	28 (82%)	9 (50%)	**.033**^a^
*B. cenocepacia* ET-12	2 (6%)	0 (0%)	.54^b^
BCC	4 (12%)	2 (11%)	1.0^b^
Diabetes^e^			
CFRD	9 (27%)	2 (11%)	.51^b,h^
Type I diabetes	0 (0%)	1 (6%)	
Most recent HbA1c (mg/dL)	0.059 (0.049–0.110)	0.054 (0.046–0.090)	**.016**^c^
No. of participants on oral corticosteroids in the past 12 months prior to the testing day^e^	5 (15%)	0 (0%)	.15^b^
Most recent vitamin D level (nmol/L)	63 (23–120)	57 (19–132)	.20^c^
Hospitalization^g^			
No. of participants^e^	8 (24%)	3 (17%)	.73^b^
No. of time			
1	6 (18%)	2 (11%)	.60^c^
2	2 (6%)	1 (6%)	
Total duration (days)	0 (0–55)	0 (0–49)	.60^c^
Time spent on PA (hr/wk)	4.9 (0.5–21)	6.3 (1.1–60)	.20^c^
Time spent on strengthening exercise (hr/wk)	0.17 (0–10.67)	0 (0–3)	.27^c^

Values are presented as medians (min-max), unless otherwise indicated.

*B. cenocepacia* ET-12 *Burkholderia cenocepacia* epidemic stain multi-locus diversity analysis enzyme Electrophoretic Type 12, BCC *Burkholderia cepacia* complex, BMI Body mass index, CFRD Cystic fibrosis related diabetes, FEV_1_ Forced expiratory volume in 1 second, FVC Forced vital capacity, HbA1c Hemoglobin A1c, PA Physical activity, *P. aeruginosa Pseudomonas aeruginosa*.

^a^chi-square test.

^b^Fisher’s exact test.

^c^Mann-Witney-Wilcoxon test.

^d^Student’s t-test.

^e^Data are presented as no. of participants (% of participants).

^f^Measures were obtained on the testing day, which were highly correlated to the measures from 3 months and 6 months prior to the testing day (r = 0.93–0.98), and to the baseline (i.e. highest measure over a year period) (r = 0.91–0.95).

Data were obtained over a 12-month period prior to the testing day.

Statistic test was performed when combining CFRD and type I diabetes as there was *n*=1 for type I diabetes.

### Quadriceps muscle size

The median (min-max) RF-CSA was 10.4 cm^2^ (4.6–22.3) cm^2^ for the minimal group and 9.1 cm^2^ (2.6–13.7) cm^2^ for the residual group. The quadriceps layer thickness was 5.5 cm (3.5–8.8) cm for the minimal group and 5.3 cm (2.9–7.6) cm for the residual group.

The multivariable linear regression model for RF-CSA is summarized in Table 2. People in the residual group had smaller RF-CSA than people in the minimal group by 3.22 cm^2^ [95% CI (1.03, 5.41) cm^2^, *p*=.0049], after adjusting for oral corticosteroid use and *P. aeruginosa* colonization.

**Table 2.** Multivariable linear regression model for RF-CSA.

Variable	Beta	95% CI	*p*-value
Intercept	16.62	12.68, 20.56	<.0001
Residual function CFTR mutations	**−3.22**	**−5.41**, **-1.03**	.0049*
Oral corticosteroid use in the past 12 months	−3.53	−6.93, −0.13	.0042
*P. aeruginosa* colonization	−2.36	−4.65, −0.078	.043

Adjusted R^2^ = 0.17; * p < .05.

CFTR Cystic fibrosis transmembrane conductance regulator, *P. aeruginosa Pseudomonas aeruginosa*, RF-CSA Rectus femoris cross-sectional area.

The multivariable linear regression model for quadriceps layer thickness is summarized in Table 3. There was no significant difference in quadriceps layer thickness between the two CFTR mutation groups, after adjusting for vitamin D level, FVC % predicted, and the interaction term between vitamin D level and CFTR mutation group (*p* = .42). The relationship between quadriceps layer thickness and CFTR mutation group depended on the vitamin D level (*p* = .032). That is, when the vitamin D level was below the median level for all patients (i.e., 59 nmol/L), the residual group had larger quadriceps layer thickness than the minimal group. However, when the vitamin D level was at or above 59 nmol/L, the minimal group had larger quadriceps layer thickness.

**Table 3.** Multivariable linear regression model for quadriceps layer thickness.

Variable	Beta	95% CI	*p*-value
Intercept	0.015	−2.18, 2.21	.99
Residual function CFTR mutations	**0.53**	**−0.77, 1.83**	**.42**
Most recent vitamin D level (nmol/L)	0.04	0.0090, 0.066	.0011
FVC (% predicted)	4.27	2.86, 5.69	<.0001
Most recent vitamin D level × CFTR mutation group	−0.015	−0.035, 0.0046	.13

Quadriceps layer thickness: layer thickness of rectus femoris + vastus lateralis + the average of vastus intermedius taken anteriorly and laterally. Adjusted R^2^ = 0.47. The variance inflation factor was 7.20–13.92, which was expectably high because of the interaction.

CFTR Cystic fibrosis transmembrane conductance regulator, FVC Forced vital capacity.

### Quadriceps muscle quality

Because of differences in ultrasound settings (frequency, depth and gain) being used to obtain RF-ECHO images in 11 participants, only 41 participants (27 from the minimal group and 14 in the residual group) were included in the RF-ECHO analysis.^32^ The median (min-max) RF-ECHO was 47 (30–66) for the minimal group and 47 (38–67) for the residual group. The multivariable linear regression model for RF-ECHO is summarized in Table 4. There was no significant difference in RF-ECHO between the two CFTR mutation groups, after adjusting for age, body weight, HbA1c, and *P. aeruginosa* colonization (*p*=.19).

**Table 4.** Multivariable linear regression model for RF-ECHO.

Variable	Beta	95% CI	*p*-value
Intercept	49.06	28.15, 69.98	<.0001
Residual function CFTR mutations	**5.20**	**−2.63, 13.03**	**.19**
Age (year)	0.057	−0.30, 0.41	.75
Body weight (kg)	−0.32	−0.52, −0.12	.0029
Most recent HbA1c (mg/dL)	137.14	−90.43, 364.71	.23
*P. aeruginosa* colonization	5.02	−1.32, 11.37	.12

Adjusted R^2^ = 0.29.

CFTR Cystic fibrosis transmembrane conductance regulator, HbA1c Hemoglobin A1c, *P. aeruginosa Pseudomonas Aeruginosa*, RF-ECHO:Rectus femoris echogenicity.

### *Post hoc* analyses

**Figure 2.**
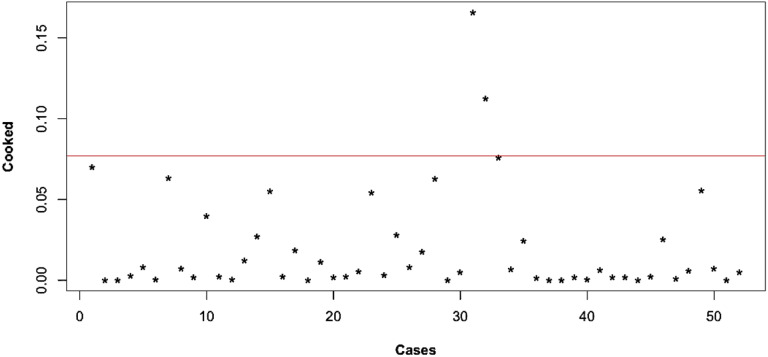
Influential cases of RF-CSA identified by Cook’s distance. The red line is the cut-off point, defined as 4/*n*, i.e. 4/52 or 0.77. RF-CSA Rectus femoris ross-sectional area.

The Cook’s distance plot for RF-CSA showed three influential cases (Figure 2). The sensitivity analyses showed that when the influential cases were excluded the difference in RF-CSA between the two CFTR mutation groups was reduced and still significant – people in the residual group had smaller RF-CSA than those in the minimal group by 1.86 cm^2^ [95% CI (0.080, 3.64) cm^2^, *p* = .041]. When the confounders for the quadriceps layer thickness were adjusted instead, the sensitivity analyses showed that the difference in RF-CSA between the two CFTR mutation groups was insignificant, after adjusting for Vitamin D level, FVC % predicted, and the interaction term between vitamin D level and CFTR mutation group (*p* = .40).

## Discussion

To the best of our knowledge, this is the first study exploring the relationship between the severity of CFTR protein dysfunction and the extent of impairment in skeletal muscle size and quality in adults with CF. We found that adults with CF with severe CFTR protein dysfunction had larger quadriceps muscle size, as measured by RF-CSA, compared to those with mild CFTR protein dysfunction. However, the difference between the two CFTR mutation groups became insignificant when adjusting for the confounders of another measure of quadriceps muscle size – quadriceps layer thickness. This sensitivity analysis indicates that the result of RF-CSA was influenced by the specific confounders being adjusted for in the model. There was no difference in quadriceps layer thickness in people with various severities of CFTR protein dysfunction. Also, there appeared to be no difference in quadriceps muscle quality among people with different severities of CFTR protein dysfunction, although this may be affected by the reduced sample size available for analysis. Therefore, we found no differential impact of the extent of diminished CFTR protein activity on muscle size or quality.

In the comparisons of quadriceps muscle size among people with various severities of CFTR protein dysfunction, the results were contradictory to our hypotheses. From a pathophysiological point of view, a cellular/molecular study showed that mice with CFTR deficiency had a higher level of pro-inflammatory gene expression.^1^ The study also showed that when CFTR deficient mice were infected with *P. aeruginosa* (and thus stimulated an inflammatory environment) there was an increased induction of cachexia-inducing components of the ubiquitin-proteasome pathway, when compared to wild-type controls.^1^ A previous clinical study also showed that adults with CF have significantly higher pro-inflammatory cytokines like C-reactive protein, neutrophil elastase-α_1_-antiproteinase complex and tumour necrosis factor-α, compared to healthy controls.^33^ These pro-inflammatory cytokines are associated with inflammation, and are responsible for an increased protein degradation through the ubiquitin-proteasome system, resulting in muscle atrophy.^34^ Based on this literature, we hypothesized that people with CF with severe CFTR protein dysfunction would have more severe muscle atrophy compared to those with mild CFTR protein dysfunction. However, our findings on muscle size measured by quadriceps layer thickness showed no significant difference, and muscle size measured by RF-CSA showed that the difference between the two CFTR mutation groups narrowed down when the influential cases were excluded, and the difference became insignificant once the model was adjusted for a different set of confounders – the confounders of quadriceps layer thickness (another measure of quadriceps muscle size). Therefore, the sensitivity analysis indicated that the set of confounders being adjusted for had an impact on the results of RF-CSA. Although the confounders to be considered were selected a priori based on previous literature, the confounders included in different models in this study were determined by a vigorous statistic process, the purposeful selection technique, in this study, which was a data-driven approach. For example, even though there is a significant difference in the age between the two CFTR mutation groups (see Table 2) and age is a major contributing factor of skeletal muscle size and contractile function,^20^ age did not exert enough impact on the variables in the study cohort and thus was not selected by the prospective selection technique the RF-CSA and quadriceps layer thickness models. Future studies with a different set of data may lead to different sets of confounders to be adjusted for, resulting in different conclusions. More studies are needed to derive a firm conclusion.

While both RF-CSA and quadriceps layer thickness represent quadriceps muscle size, the results presented here in the comparison between the two CFTR mutation groups differed. The RF-CSA is a two-dimensional measure, capturing all the sarcomeres – the functional unit of muscle contraction – of the RF, whereas quadriceps layer thickness is a one-dimensional measure, including three quadriceps muscles – RF as well as VL and VI. Therefore, although they both were measures of quadriceps muscle size, they were measuring different dimensions and included different muscles. This may be the reason for different results for quadriceps muscle size obtained in this study.

We were unable to find differences in muscle quality among people with different severities of CFTR protein dysfunction. In a study with people in the critical care setting, nutritional and exercise-focused interventions were shown to help maintain muscle quality (and prevent muscle wasting).^35^ In our study cohort, the adults with CF were mostly well-nourished, and all participants were engaged in some PA, with some in strengthening exercise, which may help maintain the integrity of muscle quality, despite CFTR protein dysfunction.

There are some limitations to note for this study. First, this study did not reach the intended sample size because of the COVID-19 lockdown. It is possible that we were underpowered to detect some of the relationships in this study. Second, our RF-ECHO data for analysis were incomplete because different ultrasound settings were used for some participants. The inter-tester mean difference of the RF-ECHO was also relatively high, although the testers were randomly assigned to test participants to reduce bias. Therefore, the RF-ECHO results should be interpreted with caution. Third, PA level was measured using a self-reported questionnaire, which is subject to recall bias. An objective measure of PA by accelerometery would offer additional and more in-depth insight, e.g. greater resolution of intensity thresholds and patterns of behaviour. Also, information obtained on the strengthening exercise from the 7D-PAR only included duration. In future studies, it would be more informative to obtain more specific data on participants’ strengthening exercise routine, e.g. upper versus lower limb strengthening exercise, and the intensity, as these may have impacts on muscle size and muscle quality.

In conclusion, although people with severe CFTR protein dysfunction appeared to have larger RF-CSA, this was influenced by the confounders being adjusted for in the model. There was no difference in the quadriceps layer thickness among people with various severities of CFTR protein dysfunction. Also, there appeared to be no difference in muscle quality (i.e. the amount of infiltration of intramuscular adipose or fibrous tissue) among people with different severities of CFTR protein dysfunction although this may be affected by the reduced sample size available for analysis. Therefore, there do not appear to have any differential impact of the extent of diminished CFTR protein activity on quadriceps muscle size or quality in our study cohort. Based on these findings, CFTR mutation status cannot be used to differentiate leg muscle size or quality in people with CF.
